# Traditional Indian Medicines Used for the Management of Diabetes Mellitus

**DOI:** 10.1155/2013/712092

**Published:** 2013-06-05

**Authors:** Syed Ibrahim Rizvi, Neetu Mishra

**Affiliations:** ^1^Department of Biochemistry, University of Allahabad, Allahabad 211002, India; ^2^Centre of Food Technology, University of Allahabad, Allahabad 211002, India

## Abstract

Plants have always been a source of drugs for humans since time immemorial. The Indian traditional system of medicine is replete with the use of plants for the management of diabetic conditions. According to the World Health Organization, up to 90% of population in developing countries use plants and its products as traditional medicine for primary health care. There are about 800 plants which have been reported to show antidiabetic potential. The present review is aimed at providing in-depth information about the antidiabetic potential and bioactive compounds present in *Ficus religiosa, Pterocarpus marsupium, Gymnema sylvestre, Allium sativum, Eugenia jambolana, Momordica charantia*, and *Trigonella foenum-graecum*. The review provides a starting point for future studies aimed at isolation, purification, and characterization of bioactive antidiabetic compounds present in these plants.

## 1. Introduction

Diabetes mellitus is a growing problem worldwide entailing enormous financial burden and medical care policy issues [1]. According to International Diabetes Federation (IDF), the number of individuals with diabetes in 2011 crossed 366 million, with an estimated 4.6 million deaths each year [2]. The Indian subcontinent has emerged as the capital of this diabetes epidemic. The reported prevalence of diabetes in adults between the ages of 20 and 79 is as follows: India 8.31%, Bangladesh 9.85%, Nepal 3.03%, Sri Lanka 7.77%, and Pakistan 6.72% [3]. 

Indians show a significantly higher age-related prevalence of diabetes when compared with several other populations [4]. For a given BMI, Asian Indians display a higher insulin level which is an indicator of peripheral insulin resistance. The insulin resistance in Indians is thought to be due to their higher body fat percentage [5, 6]. Excess body fat, typical abdominal deposition pattern, low muscle mass, and racial predisposition may explain the prevalence of hyperinsulinemia and increased development of type 2 diabetes in Asian Indians. 

Diabetes is characterized by metabolic dysregulation primarily of carbohydrate metabolism, manifested by hyper-glycemia resulting from defects in insulin secretion, impaired insulin action, or both [7]. Uncontrolled diabetes leads to a plethora of complications affecting the vascular system, eyes, nerves, and kidneys leading to peripheral vascular disease, nephropathy, neuropathy, retinopathy, morbidity, and/or mortality. 

According to the World Health Organization (WHO), up to 90% of the population in developing countries uses plants and its products as traditional medicine for primary health care [8]. The WHO has listed 21,000 plants, which are used for medicinal purposes around the world. Among these, 2500 species are in India [9]. There are about 800 plants which have been reported to show antidiabetic potential [10]. A wide collection of plant-derived active principles representing numerous bioactive compounds have established their role for possible use in the treatment of diabetes [10].

The most common and effective antidiabetic medicinal plants of Indian origin are Babul (*Acacia arabica), *bael* (Aegle marmelose), *church steeples* (Agrimonia eupatoria), *onion *(Allium cepa), *garlic* (Allium sativum),* ghrita kumara *(Aloe vera), *neem* (Azadirachta indica), * ash gourd* (Benincasa hispida),* Beetroot *(Beta vulgaris), *fever nut* (Caesalpinia bonducella), *bitter apple* (Citrullus colocynthis), *ivy gourd* (Coccinia indica), *eucalyptus* (Eucalyptus globules), *banyan tree* (Ficus benghalenesis)*, gurmar *(Gymnema sylvestre), *gurhal* (Hibiscus rosa-sinesis)*, sweet potato* (Ipomoea batatas), *purging Nut* (Jatropha curcas)*, mango* (Mangifera indica), *karela* (Momordica charantia), *mulberry* (Morus alba), *kiwach* (Mucuna pruriens), *tulsi* (Ocimum sanctum),* bisasar *(Pterocarpus marsupium), *anar* (Punica granatum), *jamun* (Syzygium cumini), *giloy* (Tinospora cordifolia), *and methi* (Trigonella foenum-graecum).* All these plants are a rich source of phytochemicals.

The present review presents the antidiabetic efficacy of some important plants used in traditional system of medicine in India for the management of type 2 diabetes mellitus.

## 2. Indian Medicinal Plants with Antidiabetic Potential

### 2.1. *Ficus religiosa*

*Ficus religiosa*, commonly known as peepal in India, belongs to family Moraceae. *Ficus religiosa* has been reported to be used in the traditional system of Ayurveda for the treatment of diabetes [11]. *F. religiosa* has been shown to possess a wide spectrum of *in vitro* and *in vivo* pharmacological activities: antidiabetic, hypolipidemic, anticonvulsant, anti-inflammatory, analgesic, antimicrobial, antiviral, antioxidant, antitumor, antiulcer, antianxiety, anthelmintic, antiasthmatic, immunomodulatory, estrogenic, endothelin receptor antagonist, apoptosis inducer, cognitive enhancer, and antihypertensive [12].

Decoction prepared from the bark is used in treatment of diabetes [13]. The plant is believed to contain several bioactive principles including tannins, saponins, polyphenolic compounds, flavonoids, and sterols. Sitosterol-d-glucoside present in the bark of *Ficus religiosa* is believed to elicit hypoglycemic activity in rabbits [14]. The bioactive components present in *Ficus *are leucocyandin 3-O-beta-d-galactosyl cellobioside, leucopelargonidin-3-O-alpha-L rhamnoside [15, 16]. The phytoconstituents present in *Ficus* can impart a significant antidiabetic effect. It has been reported to contain phytosterols, flavonoids, tannins, and furanocoumarin derivatives, namely, bergapten and bergaptol [17].

The leaves of *Ficus religiosa* have also been studied for antihyperglycemic activity [18]. Oral incorporation of aqueous extract of *Ficus religiosa* for 21 days caused a significant lowering in blood glucose levels, and an elevated level of insulin has been observed. The skeletal muscle is an important site for insulin-stimulated glucose uptake. Decrease in muscle and hepatic glycogen in diabetes was observed to be corrected by peepal extract [19, 20]. 

Secondary complications of diabetes that is hypercholesteremia and hypertriglyceridemia were found to decrease through significantly reduced serum triglycerides and total cholesterol levels in STZ-diabetic rats [21]. Administration of aqueous extract of bark at the dose of 500 mg/kg has been reported to ameliorate blood glucose level, hepatic enzymes, and lipid parameters in streptozotocin-induced diabetic rats [22].

Oxidative stress is one of the major etiologies in the pathogenesis and complications of type 2 diabetes. *F. religiosa* has been reported to modulate the enzymes of antioxidant defence system to combat oxidative stress. Restoration of glutathione and inhibition of malondialdehyde content has shown the antioxidative property of *Ficus religiosa* [23]. 

### 2.2. *Eugenia jambolana*

*Eugenia jambolana* (black plum or jamun) belongs to the family Myrtaceae. The most commonly used plant parts are seeds, leaves, fruits, and bark.* Eugenia jambolana* is an evergreen tropical tree of 8 to 15 m height, with smooth, glossy turpentine-smelling leaves. The bark is scaly gray, and the trunk is forked. There are fragrant white flowers in branched clusters at stem tips and purplish-black oval edible berries. The berries contain only one seed. The taste is generally acidic to fairly sweet but astringent. This tree is known to have grown in Indian subcontinent and in other regions of South Asia such as Nepal, Burma, Sri Lanka, Indonesia, Pakistan, and Bangladesh from ancient time.

Jamun has been reported to be used in numerous complementary and alternative medicine systems of India and, before the discovery of insulin, was a frontline antidiabetic medication even in Europe. The brew prepared by jamun seeds in boiling water has been used in the various traditional systems of medicine in India [24].

*Eugenia jambolana* is one of the widely used medicinal plants in the treatment of diabetes and several other diseases. The plant is rich in compounds containing anthocyanins, glucoside, ellagic acid, isoquercetin, kaempferol, myricetin, and hydrolysable tannins (1-0-galloyl castalagin and casuarinin). The seeds also contain alkaloid jambosine and glycoside jamboline, which slows down the diastatic conversion of starch into sugar [25]. 

The whole plant of *Eugenia jambolana *is reported to show antioxidative defence due to numerous phytochemical constituents present in it. The bark of jamun is rich in several bioactive compounds including quercetin, betulinic acid, B- sitosterol, eugenin, ellagic and gallic acid [26], bergenin [27], tannins [28], and flavonoids. Fruits contain glucose, fructose, raffinose [29], malic acid [30], and anthocyanins [31]; leaves are rich in acylated flavonol glycosides [32], quercetin, myricetin, and tannins [33] all of which have hypoglycemic ability.

The blood glucose-lowering effect of *Eugenia jambolana* may be due to increased secretion of insulin from the pancreas or by inhibition of insulin degradation [34]. *Eugenia jambolana *is also reported to have lipid-lowering effect evidenced by reduction of blood cholesterol, triglycerides, and free fatty acids [35]. This effect has been reported to be due to the presence of flavonoids, saponins, and glycosides in the extract which is reported to decrease the activity of enzyme 3-HMG Co-A reductase in liver [36]. *Eugenia jambolana* seed extract is reported to reduce blood pressure probably due to the ellagic acid present in it [33].

Addition of ethanolic extract of seeds and seed powder of *Eugenia jambolana* in alloxan-induced diabetic rats showed significant reduction in blood sugar level and enhancement in the histopathology of pancreatic islets [37]. Decrease in glycosuria and blood urea levels has also been reported. Similar kind of results has also been reported in numerous studies done on dogs and rabbits [38, 39]. 

*Eugenia jambolana *fruit juice is diuretic and has been reported to provide a soothing effect on human digestive system [40]. The gastroprotective effect has also been reported in jamun seeds. Elevation of antioxidant status and mucosal defensive properties might be the possible mechanisms behind gastroprotective properties present in jamun. Presence of flavanoids in the seeds provides the gastric ulcer protective activity to jamun [40]. Jamun shows antiviral activity against goat pox and the highly pathogenic avian influenza (H5N1) virus [41, 42].

The efficacy of *Eugenia jambolana* has also been tested in preclinical and clinical studies [43, 44] for hypolipidemic [45], anti-inflammatory, [46], neuropsychopharmacological [47], antiulcer, [48], antibacterial [49], anti-HIV [50], antidiarrhoeal [49], and antihypertensive activities [47].

### 2.3. *Momordica charantia*

*Momordica charantia *(bitter gourd or karela) belongs to the family Cucurbitaceae. Fruit as a whole and fruit's seeds are the parts most frequently used for therapeutic benefits. *Momordica charantia* is a popular fruit used for the treatment of diabetes, cardiovascular diseases, and related conditions amongst the indigenous population of Asia, South America, and East Africa. It is often used as a vegetable in diet. Bitter gourd contains bioactive substances with antidiabetic potential such as vicine, charantin, and triterpenoids along with some antioxidants [51]. Several preclinical studies have documented the antidiabetic and hypoglycaemic effects of *Momordica charantia* through various hypothesised mechanisms [52].

Several studies have demonstrated antibacterial, antiviral, anticancer, and antidiabetic activities, in *Momordica charantia* [53, 54]; however, the antidiabetic activity has been widely reviewed. In several animal studies, bitter gourd has been reported to ameliorate the metabolic syndrome, where diabetes is one of the risk factors [55–57]. In a study conducted on Taiwanese adults, a significant reduction in waist circumference, improvement in diabetes, and symptoms of metabolic syndrome has been observed [58].

The hypoglycemic and lipid-lowering properties of bitter melon have been observed [59]. Studies have shown that *Momordica charantia *can repair damaged β-cells thereby stimulating insulin levels [60] and also improve sensitivity/signalling of insulin [57]. Bitter gourd is also reported to inhibit absorption of glucose by inhibiting glucosidase and suppressing the activity of disaccharidases in the intestine [61]. 

Ethanolic extract of *Momordica charantia* is reported to show antihyperglycemic effect in normal and streptozotocin diabetic rats which might be due to inhibition of glucose-6-phosphatase and also stimulation of the activity of hepatic glucose-6-phosphate dehydrogenase [62]. Studies have reported that triterpenoids may be the hypoglycemic components present in karela which could be responsible for activation of AMP-activated protein kinase [63]. The blood glucose-lowering activity of karela has been reported in several animal models [64]. 

Bitter melon is also effective in loosening adiposity. It is reported to decrease the weight of epididymal and retroperitoneal white adipose tissues [54]. Bitter melon is found effective in augmenting skeletal muscle strength, an effect which could be due to higher mRNA expression for the glucose transporter 4 [55]. Extracts/fractions of *Antidesma madagascariense* and *Momordica charantia* were found to significantly inhibit the activity of *α*-glucosidase, a key carbohydrate hydrolyzing enzyme. However, glycogen-loaded mice showed significant depressive effect on increasing the level of postprandial blood glucose after ingestion of *Momordica charantia *[65]. Presence of saponins to some extent might justify the inhibitory activities on *α*-amylase and *α*-glucosidase. Saponins are also supposed to stimulate insulin secretion [66].

### 2.4. *Ocimum sanctum*

*Ocimum sanctum* L. (holy basil or tulsi) belongs to the family Lamiaceae. Every part of the plant is used as a therapeutic agent against several diseases. *Ocimum* (holy basil) is reported to grow worldwide. Nutritional and chemical composition of holy basil makes it a plant with immense potential. Eugenol, the active constituent present in *O. sanctum* L., has been found to be responsible for its therapeutic potential [67]. Major bioactive constituents present in the leaves and stems of holy basil include flavonoids, saponins, tannins, triterpenoids, rosmarinic acid, apigenin, isothymusin, isothymonin, cirsimaritin, orientin, and vicenin. Tulsi leaves oil contains eugenol, ursolic acid, carvacrol, linalool, limatrol, and caryophyllene along with eugenol. Seeds oil is known to have fatty acids and sitosterol while seed mucilage contains some sugars. Anthocyanins are present in green leaves. Furthermore, tulsi is also rich in vitamins, minerals, chlorophyll, and many other phytonutrients.

Antidiabetic properties of tulsi were appreciated in Ayurveda [68]. A significant reduction in blood glucose, glycosylated hemoglobin, and urea along with a simultaneous increase in glycogen, hemoglobin, and protein in streptozotocin-induced diabetic rats has been observed when rats were supplemented with ethanolic extract of *O. sanctum* [69]. Leaf extract of *O. sanctum* L has been reported to stimulate the physiological pathways of insulin secretion [70]. *O. sanctum* L. showed serum glucose-lowering effect when the extract was given to normal rats for 30 days [71].* O. sanctum* L. is reported to reduce the serum level of cortisol and glucose in male mice showing its antiperoxidative effect [72].

Studies have reported that oral administration of alcoholic extract of leaves of *O. sanctum* L. significantly reduced blood sugar level in normal, glucose-fed hyperglycemic, and streptozotocin-induced diabetic rats. Improvement in the action of exogenous insulin in normal rats has also been recorded [73]. Mixed extract of *P. marsupium* and *O. sanctum* has been recorded to not only rectify dyslipidemia but also restore the endogenous antioxidant levels in alloxan-induced diabetic rats [74].

Chloroform extracts of aerial parts of tulsi have been able to ameliorate the derangements in lipid metabolism caused due to diabetes mellitus in alloxan-induced diabetic rats. The extract significantly decreased elevated level of serum glucose and also reversed the cholesterol, triglyceride, and LDL values [75].

The hydroalcoholic extract of *O. sanctum* L. given to stress-induced male Wister rats is reported to significantly prevent the chronic resistant stress induced rise in plasma cAMP level, myocardial superoxide dismutase, and catalase activities [76]. Ursolic acid isolated from *O. sanctum* L. has been reported to protect heart cells from Adriamycin-induced lipid peroxidation [77]. *O. sanctum* L is also used to control blood cholesterol. A marked decrease in serum cholesterol, triacylglycerol, and LDL + VLDL cholesterol as compared to untreated cholesterol-fed group was observed in cholesterol-fed rabbits when supplemented with *O. sanctum* L. seed oil for four weeks [78]. A similar kind of study performed on normal albino rabbits showed lowered levels of serum total cholesterol, triglyceride, phospholipids, and LDL-cholesterol and a significant boost in the HDL-cholesterol and total fecal sterol contents with incorporation of fresh leaves of tulsi [79].

Along with antidiabetic and cardioprotective effects, *O. sanctum* L. has also been suggested to acquire antifungal [80], antimicrobial [81], analgesic [82], anthelmintic [83], antistress [9], antifertility [84], anti-inflammatory [85], antioxidant [78, 86], gastroprotective [87], immunomodulatory [88], antithyroidic [89], anticancer [90], and radioprotective effects [91, 92]. Tulsi is reported to provide a protection for central nervous system [93] and against sexually transmitted diseases [94].

### 2.5. *Pterocarpus marsupium*

*Pterocarpus marsupium *(indian kino tree, bijasar) belongs to the family Fabaceae. Plant parts used most commonly are heart wood, leaves, flowers, bark, and gum. *Pterocarpus marsupium* grows very well in India, Nepal, and Sri Lanka. As per Ayurveda, it is one of the most versatile medicinal plants with a wide spectrum of biological activities. Every part of the tree has been acknowledged for its therapeutic potential. This tree grows up to 30 metres in height. Compositional studies on bijasar have shown this plant to be a good source of polyphenols. *P. marsupium* contains terpenoids and phenolic compounds: β-sitosterol, lupenol, aurone glycosides, epicatechins, and iso-flavonoids [95, 96].

*P. marsupium* is known for its antidiabetic activity [97]. Besides eliciting a strong antidiabetic property, *Pterocarpus marsupium* is reported to be effective against several diseases. It is reported to be antiobesity, antihyperlipidemic [98], anti-inflammatory, anthelmentic [99, 100], antioxidative, antitumorigenic and antiulcerative [71, 101]. 

*Pterocarpus marsupium* is reported to have not only hypoglycemic property but also β-cell protective and regenerative properties [102], effects which have been attributed to the flavonoid content in the plant. Complete restoration of normal insulin secretion and regeneration of beta cells have been reported in various experimental models of diabetes [103, 104]. A methanolic extract of *Pterocarpus marsupium* when supplemented for 7 and 14 days to STZ-diabetic rats showed normalization of streptozotocin-distressed serum glucose by correcting glycosylated hemoglobin (HbA1c), serum protein, insulin, alkaline and acid phosphatase, and albumin levels [105]. 

The blood sugar-lowering activity has been endorsed to be due to the presence of tannates in the extract of the plant. Antihyperlipidemic activity is contributed probably to the marsupin, pterosupin, and liquiritigenin present in the plant [106]. (−) Epicatechin has been shown to have insulinogenic property by enhancing insulin release and conversion of proinsulin to insulin. (−) Epicatechin has also been shown to possess insulin-like activity [107, 108]. Epicatechin has also been shown to strengthen the insulin signalling by activating key proteins of that pathway and regulating glucose production through AKT and AMPK modulation in HepG2 cells [109].

### 2.6. *Trigonella foenum-graecum*

*Trigonella foenum*-*graecum* (fenugreek, methi) belongs to the family Fabaceae. Seeds and leaves are the most frequently used parts of the plant. *Trigonella foenum*-*graecum* L. (fenugreek) is cultivated throughout India and in some other parts of the world as a semiarid crop [80]. It is used both as a vegetable and as a spice in India. Fenugreek is well known for its pungent aromatic properties, and it is a flavoring agent in food [110]. Studies on different experimental models have proved that fenugreek has strong antidiabetic properties [111, 112]. Human studies have also confirmed the glucose and lipid-lowering ability of fenugreek [113].

Several studies have demonstrated that fenugreek seed extract, mucilage of seeds, and leaves can decrease blood glucose and cholesterol levels in humans and experimental diabetic animals [114, 115]. The therapeutic potential of fenugreek is primarily due to the presence of saponins [116], 4-hydroxyisoleucine [117], and trigonelline, an alkaloid [118] and a high-fiber content [119].

The antihyperglycemic effect has been correlated with decline in somatostatin and high plasma glucagon levels [120]. Fenugreek seed powder has been shown to normalize the activity of creatinine kinase in liver, skeletal muscles, and heart of diabetic rats [121]. The antihyperglycemic effect of fenugreek has been hypothesized to be due to the amino acid 4-hydroxyisoleucine which acts by the enhancement of insulin sensitivity and glucose uptake in peripheral tissues [122]. The steroids present in methi have been reported to reduce blood glucose level when supplemented to diabetic rats [123]. A considerable increment of the area of insulin-immunoreactive β cells has been observed [124]. 

A study on intestinal and renal disaccharidases activity in STZ-induced diabetic rats proved the beneficial effects of fenugreek seed mucilage by enhancing the reduction in maltase activity during diabetes [125]. The optimistic influence of fenugreek supplementation on intestinal and renal disaccharidases has been reported [126]. A marked reduction in renal toxicity has been observed when fenugreek oil is incorporated in the diet of alloxanized rats [125]. 

### 2.7. *Gymnema sylvestre*

*Gymnema sylvestre* (gurmar) belongs to the family Asclepiadaceae. It is a herb native to the tropical forests of India and Sri Lanka.* G. sylvestre* is a large climber, with roots at nodes. It is a potent antidiabetic plant used in ayurvedic preparations. Several studies have proved its antidiabetic potential in animal models [125]; when combined with acarbose it is reported to reduce intestinal transport of maltose in rats [127]. Absorption of free oleic acid in rats has also been reduced [128]. 

Aqueous extract of *G. sylvestre* has been reported to cause reversible increases in intracellular calcium and insulin secretion in mouse and human β cells with type 2 diabetes [129]. Regeneration of the cells in the pancreas might raise the insulin levels [130]. *G. sylvestre* can also help prevent adrenal hormones from stimulating the liver to produce glucose in mice, thereby reducing blood sugar levels [131]. A group of triterpene saponins, known as gymnemic acids and gymnemasaponins are found to be present in *G. sylvestre* which are responsible for the reported pharmacological properties.

Oral administration of *Gymnema* is reported to be effective against chronic inflammation [132], obesity [133, 134], and pancreatic β cell dysfunction [135]. *G. sylvestre* suspension shows tremendous diabetic potential against alloxan-induced diabetic albino male rats [136]. The hypoglycemic effect of ethanolic extract of *G. sylvestre* is reported to be due to enhanced effect of insulin which comes into play by increasing either the pancreatic secretion of insulin from β cells or its release from the bound form [130, 137, 138]. A significant correlation between the good glycemic control and phospholipid levels has been observed [139]. Oral administration of *G. sylvestre* to rats has been reported to result in increased utilization of glucose and/or by decreasing mobilization of fat [136]. A significant reduction in body weight, plasma proteins, and total hemoglobin levels has also been observed [136].

### 2.8. *Allium sativum*

*Allium sativum *(garlic) commonly called lahsun belongs to the family Amaryllidaceae. Leaves and bulb are the parts frequently used. As per Ayurveda it is a miraculous plant used against a variety of problems including insect bites, intestinal worms, headache, and tumors [140]. Garlic is also used in folk medicine for the management of cardiac diseases, cancer, parasitic, fungal diseases, and diabetes [141, 142]. The principle bioactive components present in garlic are allicin, allixin, ajoene, and other organosulphur compounds.

 Biological and therapeutic functions of garlic are basically due to the organosulphur compounds they possess [143]. These chemical components are thought to exhibit numerous biological effects including lowering of cholesterol and glucose, cancer prevention, and antimicrobial properties [144]. Studies have proved that the consumption of garlic significantly decreased fasting blood sugar levels [145]. Diallyl trisulfide has been proved to improve glycemic control in STZ-induced diabetic rats. [146] Incorporation of garlic juice resulted in better utilization of glucose in glucose tolerance tests performed in rabbits, while allicin at a dose of 250 mg/kg was 60% as effective as tolbutamide in alloxan-induced diabetic rabbits [147].

Garlic may act as an antidiabetic agent by increasing either the pancreatic secretion of insulin from the β cells or the release of bound insulin [148]. Allicin is supposed to enhance serum insulin by combining with cysteine and sparing it from SH group reactions [147]. The beneficial effects of N-acetylcysteine, an organosulfur from allium plants, on serum lipids and glucose are related to its antioxidant property. N-Acetylcysteine is reported to reduce the oxidative stress by improving the endogenous antioxidant defences [149]. 

Allicin, a sulfur-containing compound, is responsible for the pungent flavour and significant hypoglycemic activity in garlic. This effect is supposed to be due to enhanced hepatic metabolism, release of insulin, and/or insulin-sparing effect [150, 151]. S-allyl cystein sulfoxide the precursor of allicin is reported to control lipid peroxidation and hyperglycemia in rats [152]. 

Cardiovascular complications of diabetes are reported to be prevented by the consumption of garlic [153]. Saponins are reported to reduce serum cholesterol levels [154]. Garlic juice has been found to exert antioxidant and antihyperglycemic effects in alloxan-induced diabetic rats [155].

Phytochemicals present in garlic also show antioxidative property evidenced by scavenging of reactive oxygen species [156] and increasing cellular antioxidant enzymes: superoxide dismutase, catalase, and glutathione peroxidase [157]. Garlic alone and with ginger and turmeric when tested against oxidative stress in streptozotocin (STZ)-nicotinamide diabetic rats showed 80–97% increment in the signs of hyperglycaemia and dyslipidaemia, 26–37% increase in the production of insulin and enrichment in the antioxidant defence system along with a 60–97% decrease in lipid peroxidation [158]. Administration of raw garlic homogenate was found to normalise both hepatic TBARS and GSH levels and also improve insulin sensitivity and oxidative stress in fructose-fed rats [159]. Numerous studies report that aged garlic extract inhibit the generation of glycation-derived free radicals and AGEs *in vitro*. S-Allyl cysteine, one of the bioactive ingredients of aged garlic, is a known antioxidant that possesses the capacity to inhibit AGEs synthesis [160].

## 3. Conclusion

As per Ayurveda, there exists a huge collection of plants with antidiabetic potential. Only few of them have been scientifically proven and a lot more have yet to be explored and proved. *Ficus religiosa, Gymnema sylvestre, Allium sativum, Trigonella foenum graecum, Pterocarpus marsupium, Ocimum sanctum, Momordica charantia, Eugenia jambolana,* and *Ficus religiosa *have shown varying degrees of hypoglycemic activity. These plants have also been reported to contribute in control of complications of diabetes. Future studies may target isolation, purification, and characterization of bioactive compounds present in these plants. The outcome of such studies may provide a starting point for development of potential antidiabetic drugs. This review may be helpful in the management of diabetes.
